# Autoimmune Peripheral Neuropathies and Contribution of Antiganglioside/Sulphatide Autoantibody Testing

**DOI:** 10.31138/mjr.31.1.10

**Published:** 2020-03-31

**Authors:** Dirk Roggenbuck, Emilien Delmont, Dirk Reinhold, Peter Schierack, Karsten Conrad, Joseph Boucraut

**Affiliations:** 1 Faculty of Health Sciences, Joint Faculty of the Brandenburg University of Technology Cottbus – Senftenberg, the Brandenburg Medical School Theodor Fontane and the University of Potsdam, Germany,; 2 Institute of Biotechnology, Faculty Environment and Natural Sciences, Brandenburg University of Technology Cottbus Senftenberg, Senftenberg, Germany,; 3 Referral Center for Neuromuscular Diseases and ALS, La Timone Hospital, AP-HM, Marseille France,; 4 Institute of Molecular and Clinical Immunology, Otto-von-Guericke University Magdeburg, Magdeburg, Germany,; 5 Institute of Immunology, Medical Faculty of the Technical University Dresden, Dresden, Germany,; 6 Aix Marseille Université, Institut de Neurosciences de la Timone, Medicine Faculty, Marseille, France,; 7 Immunology laboratory, Conception Hospital, AP-HM, Marseille, France

**Keywords:** immune-mediated polyneuropathy, ganglioside, sulphatide, autoantibody

## Abstract

Peripheral immune-mediated polyneuropathies (IMPN) are a diverse group of rare neurological illnesses characterized by nerve damage. Leading morphological features are mostly nerve fibre demyelination or combination of axonal damage and demyelination. There has been remarkable progress in the clinical and electrophysiological categorization of acute (fulminant, life-threatening) and chronic (progressive/remitting-relapsing) immune-mediated neuropathies recently. Besides electrophysiological and morphological makers, autoantibodies against glycolipids or paranodal/nodal molecules have been recommended as candidate markers for IMPN. The progress in testing for autoantibodies (autoAbs) to glycolipids such as gangliosides and sulfatide may have significant implications on the stratification of patients and their treatment response. Thus, this topic was reviewed in a presentation held during the 1st Panhellenic Congress of Autoimmune Diseases, Rheumatology and Clinical Immunology in Portaria, Pelion, Greece. For acute IMPN, often referred to as Guillain-Barré syndrome and its variants, several serological markers including autoAbs to gangliosides and sulphatide have been employed successfully in clinical routine. However, the evolution of serological diagnosis of chronic variants, such as chronic inflammatory demyelinating polyneuropathy or multifocal motor neuropathy, is less satisfactory. Serological diagnostic markers could, therefore, help in the differential diagnosis due to their assumed pathogenic role. Additionally, stratification of patients to improve their response to treatment may be possible. In general, a majority of patients respond well to causal therapy that includes intravenous immunoglobulins and plasmapheresis. As second line therapy options, biologicals (e.g., rituximab) and immunosuppressant or immunomodulatory drugs may be considered when patients do not respond adequately.

## Glycolipids as autoantigenic targets

Among antiglycolipid autoantibodies (autoAbs), those directed to gangliosides have been investigated best in the context of autoimmune nerve illnesses.^1 –3^ The occurrence of autoAbs to glycolipids is often preceded by infections in particular intestinal ones (*Campylobacter [C.] jejuni*). Hence, molecular mimicry has been considered as most common reason for the development of a tolerance break to glycolipids in the context of IMPN pathophysiology.^4^ There is, for instance, a striking similarity of glycan structures present on *C. jejuni* lipo-oligosaccharides with gangliosides including GM1, GM2, GD1a, GT1a and GD3.^5^

Interestingly, gangliosides also represent tumour-associated antigens often overexpressed in distinct malignancies, against which the immune system can exert effector mechanisms in the context of tumour surveillance and corresponding antitumor responses.^6^ The assumption that a tolerance break against gangliosides can be part of antitumor responses during tumorigenesis is an interesting topic; however, it is beyond the scope of this review.

**Figure 1.**
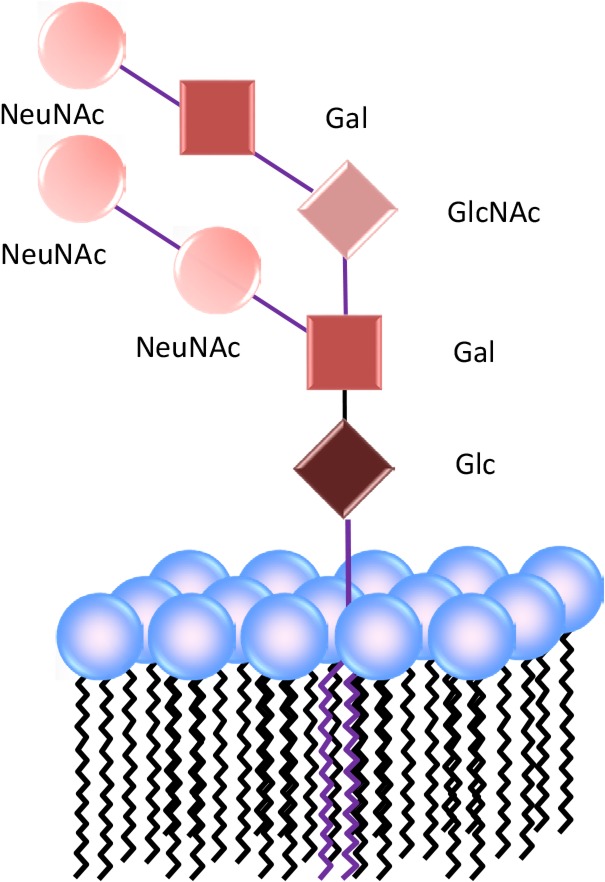
Localization of the ganglioside GT1b in the outer leaflet of the plasma membrane of nerve cells. The ceramide moiety is hidden in the outer phospholipid layer of the membrane and presumably surrounded by phospholipids, cholesterol and transmembrane molecules forming a lipid raft. Gal: galactose; Glu: glucose; GluNAc: N-acetyl glycosamine; NeuNAc: N-acetyl neuraminic acid (sialic acid)

The term ganglioside introduced by Ernst Klenk in 1942 is a combination of the two terms *ganglion* and *glycoside*, which refers to the location and composition of these molecules, respectively.^7^ Gangliosides are integrated into the cell membrane and are probably located in lipid rafts.^8,9^ Gangliosides consist of a ceramide and an oligosaccha-ride moiety with, in general, one or more neuraminic acid residues commonly referred to as sialic acids. The ceramide part is embedded in the outer leaflet of the plasma membrane and the oligosaccharides directed to the extracellular surface (*Figure 1*).^8^ Ganglioside molecules with more than two neuraminic acid residues are mainly found in the nervous system, where they can partake in cell signal transduction events. However, they are also accessible for autoimmune attacks at special locations such as the dorsal and ventral spinal roots or the sensory and motor nerve terminals.^10^ In contrast, the larger part of the peripheral nervous system is less exposed due to the blood-nerve barrier generating “immunologically privileged” sites.^3^ Altogether, the prevalence of ganglio-sides in peripheral nerves is quite variable and there is a need for more precise data on the composition and distribution of gangliosides.^3,11^ The rare involvement of the central nervous system during a tolerance break to glycolipids is very likely caused by the blood-brain barrier limiting the crossover of autoAbs in the brain.

Since peripheral nerves exert motor, sensory and autonomic functions, the site and extent of autoimmune attack against them should have a direct impact on the characteristics of the occurring clinical symptoms in patients with IMPN. Notably, in extraneural tissues, the ganglioside content is one to two orders of magnitude lower than in the nervous system.^12^ That may explain the almost negligible effect of a specific autoimmune attack against them at these sites.

In contrast to gangliosides, sulphatide such as 3-O-sulphogalactosylceramide represent a class of glycolipids with a sulphate group instead of neuraminic acid. These sulphoglycolipids differ in their strong negative charge (pKa -1.8) from the gangliosides. In the peripheral nervous system, sulphatide is mainly found in the non-compact myelin of Schwann cells, where they make up about 4–7% of all myelin lipids and are essential for the integrity of the myelin sheath.^13^ When sulphatide is absent or attacked by autoimmune responses, the lateral loops and part of the nodes of Ranvier will be disorganized and as a result the myelin sheath may not function properly.^14^ For example, cerebroside sulphotransferase-deficient mice demonstrated paranodal disruption by juxtaparanodal voltage-gated potassium channel invasion which underscores the role of sulphatide in stabilizing the paranodal junctions.^15^ Of note, sulphatide is similar to the ganglioside GM4, which, unlike other gangliosides, is like sulphatide derived from galactosylceramide (GalCer) instead of glucosylceramide (GlcCer). Interestingly, GalCer (also referred to as galactocerebroside) was the first glycolipid to be found auto-antigenic in an animal model (experimental autoimmune neuritis in rodents).^16^ Akin to sulphatide, GM4 was also determined in the myelin, but, only at lower levels.^17^

Gangliosides and sulphatide as glycolipids lack classical T-cell epitopes, so that they can be considered as thymus-independent autoantigens. Nonetheless, humoral as well as cellular autoimmune reactions, which are directed exclusively to the carbohydrate content, can be observed in humans.^18^ The occurrence of antiganglioside autoAbs in neurological illnesses was reported first in the early 1980s.^19^ These autoAbs were mainly IgM paraproteins with simultaneous reactivity to the glycan portion of the myelin-associated protein (MAG). Since the 1980s, autoAbs against more than 20 different gangliosides have been associated with a variety of acute and chronic peripheral neuropathies.^1^ The specificity of the autoAb binding to gangliosides seems to be largely defined by the number and location of the sialic acid residues and their interaction with adjacent molecules in the lipid rafts. Numerous combinations have been reported which can enhance or diminish autoAb binding.^3^ As referred to above, these differently sialylated glycosphingolipids can be located at differing sites in the peripheral nervous system with varying accessibility and even epitope presentation. Consequently, a specific autoimmune response to these epitopes can cause distinct clinical symptoms. For example, the ganglioside GM1 is highly expressed on the membranes of nerves, Schwann cells and the node of Ranvier.^20^ Thus, binding of anti-GM1 autoAbs can trigger immune responses such as complement activation with ensuing disruption of sodium channel clusters, which leads to conduction disorders and, interestingly, to impairment of motor functions in particular.^21–23^ Three major epitope patterns can be recognized by autoAbs directed to GM1, including the monospecific binding to a unique GM1 epitope, the recognition of a common sialylated epitope found on GM1 and GM2 as well as an obviously non-sialylated epitope present on GM1, GD1b and asialo-GM1.^24–26^ Furthermore, patients suffering from chronic ataxic neuropathy with ophthalmoplegia, M-paraprotein, cold agglutinins, disialosyl antibodies (CANOMAD) syndrome demonstrate in accordance with the definition of the syndrome autoAbs to gangliosides with two neuraminic (sialic) acid residues found primarily in sensory nerves.^27^ Altogether, depending on the type of neuropathy, distinct antiganglioside autoAbs or even autoAb profiles can be observed.

## Pathogenic role of autoAbs to gangliosides/sulphatide

Acquired peripheral neuropathies can be attributed to several causes, including autoimmune responses. This assumption is supported by experimental evidence on passive and active animal transfer models, active immunization with nerve components and response to immunosuppressive treatment, intravenous immunoglobulin (IVIg) administration as well as plasmapheresis.^16,28–30^ In general, multifocal demyelination of nerve cells and conditions mimicking this process are considered the leading pathogenic process in IMPN currently.^31–33^ Nonetheless, the common electrophysiological examination classifies nerve fibre damage into axonal and demyelinating damage. As a rule, axonal damage is considered to be irreversible and, in general, associated with poor clinical outcome. Recently, the node of Ranvier and adjacent regions have come into the limelight as targets for autoimmune attacks leading to reversible dysfunctional saltatory conduction.^32^ This new concept of reversible conduction failure is based on electrophysiological and experimental findings of a typical “axonal” conduction failure which, however, can rapidly recover. Thus, the term nodo-paranodopathy was coined for this particular combination.^32^ However, it remains to be seen, whether this novel concept can be applied to all IMPN as for most of them a clear axonal or demyelinating phenotype was established.

Overall, humoral (immunoglobulin, complement deposits) and cellular immune responses were observed in patients with IMPN.^34^ These findings are supported by the detection of increased serum levels of anaphylatoxin C5a and terminal complement complex (C5b9) in serum and CSF of patients with chronic inflammatory demyelinating neuropathy (CIDP) in contrast to controls.^35^ Moreover, autoreactive T-cell responses against myelin epitopes have been identified in IMPN as well as CD4+ and essentially CD8+ T cells in inflammatory infiltrates in CIDP.^36–38^ Since gangliosides should be considered as thymus-independent autoantigenic targets not bearing typical T-cell epitopes, other antigen-presenting molecules than human leukocyte antigens (HLA) class I and II need to be involved. Candidates could be the CD1 family of MHC-like molecules, which have been shown to present glycolipid and lipid antigens, including sulphatide, self-lipids and microbial ones to T cells.^39–41^ Of note, glycolipids play a pivotal role in immune cell functions regulating immune responses.^42^

Gangliosides are components of the node (GM1, GD1a, GD1b) and paranodal regions (GQ1b) but also to a lesser extent of the myelin. Sulphatide is mainly located in the non-compact myelin of Schwann cells, but can be also found in the node and paranode. Both can apparently act as autoantigenic targets, triggering immune effector mechanisms, such as the formation of IgG and IgM autoAbs. However, autoAb occurrence and the putative corresponding damage sites do not always reflect clinical findings as outlined above. Although GM1 for instance is abundant in both sensory and motor nerves, the occurrence of corresponding autoAbs is clinically mainly correlated with motor phenotypes such as acute motor axonal neuropathy (AMAN) or multifocal motor neuropathy (MMN).^43 – 45^ This may indicate other factors influencing the epitope structure of gangliosides, such as interaction with further molecules present in ganglioside-containing lipid rafts, including the gangliosides themselves as mentioned earlier. Another reason could be a greater susceptibility of motor neurons to axonal damage, although the underlying processes are largely unknown.^46^ Despite the fact that autoAbs to gangliosides were the first serum autoAbs to be linked with Guillain-Barré syndrome (GBS) and their putative pathogenic potential, the clinical and electrophysiological findings still dominate the diagnosis of IMPN.^18^ In addition to the leading autoantigenic gangliosides GM1, GD1b and GQ1b, individual autoimmune humoral reactions against a variety of other gangliosides were observed in patients with peripheral neuropathies. Though there are obviously many cross-reactive responses, the specificity of distinct patient sera to only one glycolipid target is remarkable. Even single-positive patient sera for the similarly structured sulphatide and GM4 have been reported.^47,48^

## Association of autoAbs to gangliosides/sulphatide with clinical symptoms

In contrast to acute immune-mediated neuropathies, the presence of autoAbs against gangliosides and sulphatide in chronic IMPN is still a controversial issue (*Table 1*). Their diagnostic significance was only established for a minority of them. This is probably due to the fact that chronic IMPN, such as CIDP, can encompass several clinical subentities, as well as a number of atypical variants with a wide variety of clinical phenotypes and response to treatment.^31^ Thus, IgM AutoAbs against disialosyl epitopes, especially for GD1b, have been found in chronic sensory ataxic neuropathy, which is often clinically similar to CIDP.^27^ CANOMAD syndrome patients can also develop IgM autoAbs to other disialosyl ganglio-sides such as GD3, GT1b, and GQ1b.^27^ Remarkably, the majority of patients with IgM against GD1b benefited from IVIg therapy or biologicals.^49,50^ Thus, these IgM autoAbs obviously may play a pathogenic role in the development of sensory ataxia, which can also be seen in CIDP. AutoAbs to sulphatide were found mostly in IMPN with axonal damage.^51,52^ Patients demonstrating autoAbs to sulphatide had a higher rate of conduction blocks in nerve conduction studies. Thus, the impairment of primarily motor functions may be explained by the depletion of sulphatide and myelin proteins, such as neurofascin 155, in the paranodal region.^52^ Nonetheless, a demyelinating variant with a lower prevalence was reported as well.^53^ Moreover, antisulphatide autoAbs nonreactive to sulphated glucuronic acid can also be associated with predominantly sensory neuropathies.^54^

**Table 1.** Association of antiglycolipid autoantibodies (autoAbs) and their site of attack with characteristic features of peripheral immune-mediated polyneuropathies (IMPN).

**Acute peripheral IMPN**	**Target location**	**autoAb target**	**Features**
**AMAN**	Node	GM1, GM1a/b, GD1a	Primarily IgG to GM1
**AMSAN**	Node	GM1, GM1b	
**Acute ataxic neuropathy**	Node	GD1b	Sensory ataxia with preserved motor function
**MFS**	Paranode	GQ1b, GT1a	GQ1b (95% –99%), good response to treatment
**PCB**	Paranode	GT1a, GQ1b	GT1a > GQ1b
**GBS**	Myelin, node	Sulphatide/ganglioside complexes	Most frequent autoAbs in GBS
**Chronic peripheral IMPN**			
**MMN**	Node	GM1, sulphatide	Primarily IgM to GM1, sulphatide higher rate of conduction blocks
**CANOMAD**	Node	Disialosyl gangliosides	autoAbs to GD1b – good response to treatment
**Chronic ataxic neuropathy**	Node	GD1b	Sensory ataxia with preserved motor function
**CIDP**	Myelin	Sulphatide	Younger patients, typical CIDP phenotype

AMAN: acute motor axonal neuropathy, GBS variant; AMSAN: acute motor-sensory axonal neuropathy, GBS Variant; CIDP: chronic inflammatory demyelinating polyneuropathy; CANOMAD: chronic ataxic neuropathy, ophthalmoplegia, IgM paraprotein, cold agglutinin and antidisialosyl antibodies; GBS: Guillain-Barré syndrome; MFS: Miller Fisher syndrome; MMN: multifocal motor neuropathy; PCB: pharyngeal-cervical brachial variant of GBS.

Giannotta et al. reported reactivity to sulphatide in only 1% of CIDP patients.^55^ Interestingly, a recent report highlighted an elevated frequency of at least one IgM autoAb to GM1, GD1b and sulphatide in patients suffering from CIDP.^27^ Here, patients with autoAbs to sulphatide were younger and demonstrated typical manifestations of clinical symptoms characteristic for CIDP. However, there was no association with axonal degeneration, monoclonal IgM gammopathy or with positivity of autoAbs to MAG as reported earlier.^53 , 55 – 57^ Conversely, in neuropathy patients with IgM gammopathy with late onset and sensory damage, elevated titres of IgM to sulphatide were often associated with a concomitant reactivity to MAG.^56^ Of note, the so called anti-MAG neuropathy syndrome was the first autoimmune neuropathy in which the target specificity was reported in humans.^58^

On the contrary, IgM against GM1 was found in up to 60% of patients suffering from multifocal motor neuropathy, a chronic progressive motor polyneuropathy.^59^ Normally, this IgM to GM1 is not of paraprotein origin and can interact with different epitope patterns on GM1 as outlined above. Furthermore, a recent retrospective analysis found IgM autoAbs to GM1 in 46% of patients with multifocal motor neuropathy but in only 3% of CIDP patients.^60^

In acute polyneuropathies which encompass several acute variants of the GBS, stronger associations of pathogenic autoAbs with different clinical variants were established.^25,61^ Interestingly, autoAbs to sulphatide-ganglioside complexes analysed with a combinatorial glycoarray methodology constituted the largest group of anti-glycolipid autoAbs in patients with GBS.^56^

AutoAbs to GQ1b and GT1a have been found primarily in patients with the Miller-Fisher syndrome, a subtype of GBS with involvement of ocular nerves causing ophthalmoplegia.^25 , 62 – 64^ The occurrence of autoAbs to GQ1b is related to a good prognosis characterized by complete remission of clinical and electrophysiological symptoms.^1^ Moreover, the combination of anti-GQ1b/GT1a autoAbs could be found in ataxic GBS and the pharyngeal-cervical brachial variant of GBS.^65,66^

In GBS patients showing acute axonal damage (acute motor axonal neuropathy), there is a strong association with autoAbs to sialylated epitopes present on GM1a, GM1b and N-acetylgalactoseamin-GD1a.^67,68^

The most common form of the GBS, the acute inflammatory demyelinating polyneuropathy (AIDP) can also demonstrate autoAbs to gangliosides of Schwann cells though their significance is still elusive.^69^

Acute ataxic neuropathies predominantly demonstrate anti-GD1b IgG, whereas the corresponding IgM isotype is more common in chronic variants. A similar distribution has been reported for autoAbs to GM1.^65,70^

## Assay techniques for the detection of antiganglioside autoAbs

**Figure 2.**
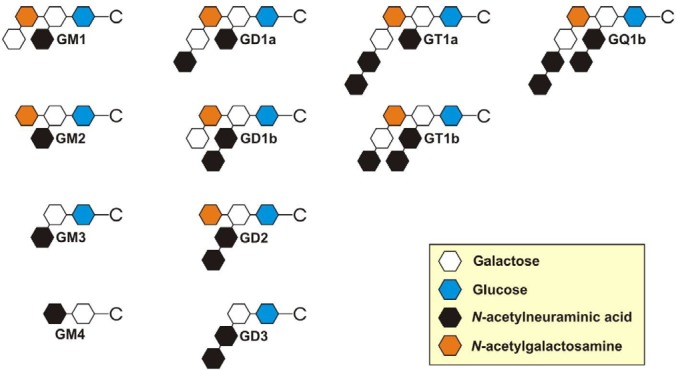
Range of gangliosides employed in multiplex line immunoassays for the detection of corresponding autoantibodies. C: ceramide

Over the last 40 years, various assay techniques have been developed for the analysis of autoAbs against gangliosides. Thin-layer chromatography overlay was one of the first antiganglioside assays to be used and is still considered the gold standard assay technique today.^27 , 71 , 72^ Due to its technical complexity, however, it is not suitable for routine use and limited to specialized laboratories only.^73^ Therefore, agglutination tests,^74^ flow cytometry analyses^75^ and several in-house^76,77^ and commercially available techniques have been developed to test for antiganglioside autoAbs.^47,77^ In particular, solid-phase enzyme immune assays have been proposed such as the enzyme-linked immunosorbent assays (ELISA) or line immunoassays (LIA). The line immunoassays (LIA) and the similar combinatorial glycoarray may be suitable alternatives, especially for the multiplex assessment of autoAbs to gangliosides or their complexes (*Figure 2*).^51,78^ The solid phase used for the immobilisation of gangliosides appears to play a pivotal role for the detection of antiganglioside autoAbs.^47^ Disease-specific antibodies presumably recognize gangliosides incorporated in cell membranes and, thus, may fail to bind to gangliosides immobilized on a non-appropriate solid phase.^77^ For example, we clearly observed that the flow cytometry technique for anti-GM1 autoAb testing was more sensitive and specific than our in-house ELISA regarding the diagnosis of motor neuropathies.^75^ However, this method is time-consuming, unsuitable for routine laboratory use and not applicable for most other gangliosides. One strategy is to use immobilized lipid complexes. By optimal preservation of the mostly conformational autoantigenic epitopes on the glycan part, and by concealing of the hydrophobic ceramide in the porous, also hydrophobic polyvinylidene difluoride membranes during the binding of the glycolipids, LIA and combinatorial glycoarray techniques may have advantages over the ELISA. The superiority of this particular hydrophobic solid phase has been demonstrated for the immobilisation of other amphiphatic molecules such as lipopolysaccharides and phospholipids exhibiting similar physicochemical characteristics.^79–83^ Hydrophobic PVDF membranes as solid phases seem to mimic membranous antigen/antibody interactions more efficiently. For example, hydrophobic membranes appear to function better to test for anti-phospholipid autoAbs in contrast to the solid phases used in enzyme-linked immunosorbent assays.^84,85^

Altogether, there are several commercially available methods for antiganglioside autoAb testing. Their advantages and drawbacks have been compared recently in depth.^77^ For this comparative analysis, patients with CANOMAD syndrome were selected according to their specific antiganglioside antibody profiles foreseen for a French national registry of CANOMAD syndrome. Line immunoassays showed the best performance and were confirmed as efficient and robust tests for the multiplex analysis of autoAbs to gangliosides.

Obviously, currently available assays differ in their accuracy, sensitivity and specificity due to the varying purity of gangliosides from different commercial sources, use of detergents in wash buffers, different incubation characteristics and background issues as a result of unspecific binding. For example, incubation at 4° C rather than room temperature appeared to be advantageous.^73^ Moreover, different cut-off values to discriminate patients from disease controls and healthy individuals influenced the assessment of serum antiganglioside autoAbs significantly.^86^ For example, a great variability in the frequency of IgM or IgG to GM1 by ELISA has been reported, either in the same laboratory or between laboratories.^86^ As mentioned above, up to 20 different autoAbs to gangliosides and sulphatide can be correlated with clinical symptoms in patients with IMPN. Thus, a key requirement is the number of autoantigenic targets since a large panel of gangliosides is essential to correctly analyse complex antiganglioside autoAb profiles found in patients with GBS or its variants, as well as with CANOMAD.^2 , 27 , 87^ Until now, the standardization and harmonization of antiganglioside autoAb testing remains an unmet requirement. Often, results obtained in different laboratories are difficult to compare. Altogether, a simple, multiplexed analysis of antiganglioside autoAbs is required for the serological processing of patients with IMPN to address the clinical need and to overcome limitations of monoplex, non-standardised immunoassays. In contrast, one of the difficulties in exploring antiglycolipid autoAbs is that they are present at low levels in healthy subjects and are often part of natural autoantibodies. Monoplex ELISAs could make it easier to determine a positive threshold for single antiglycolipid autoAbs.

It should be noted that differences in assay techniques may be the reason for different reports on the frequency of autoAbs on gangliosides and sulphatide.^55^ For example, higher frequencies of IgM autoantibodies to GM1 (16%) by LIA were found in patients with CIDP and multifocal motor neuropathy in contrast to the glycoarray technique (7%).^88,89^ In summary, there appears to be a clear need to replace in-house assays by validated, widely available tests enabling multiplex analysis of autoAbs to gangliosides and sulphatide. This development addresses the rising number of patient samples on the one hand and of newly emerging gangliosides as autoantigenic targets on the other hand in the context of the differential diagnosis of peripheral IMPN.

## Abbreviations

AMAN: Acute motor axonal neuropathy
AMSAN: Acute motor-sensory axonal neuropathy
autoAb: Autoantibody
CIDP: Chronic inflammatory demyelinating polyneuropathy
CANOMAD: Chronic ataxic neuropathy, ophthalmoplegia, IgM paraprotein, cold agglutinin and antidisialosyl antibodies
GalCer: Galactocerebroside
GBS: Guillain-Barré syndrome
IMPN: Immune-mediated polyneuropathy
IVIg: Intravenous immunoglobulin
MAG: Myelin-associated glycoprotein
MFS: Miller Fisher syndrome
MMN: Multifocal motor neuropathy
PCB: Pharyngeal-cervical brachial variant of GBS
